# Hyperpolarization-Activated Cyclic Nucleotide-Gated Ion (HCN) Channels Regulate PC12 Cell Differentiation Toward Sympathetic Neuron

**DOI:** 10.3389/fncel.2019.00415

**Published:** 2019-09-20

**Authors:** Li-Ying Zhong, Xin-Rong Fan, Zhang-Jing Shi, Zhong-Cai Fan, Jian Luo, Na Lin, Ying-Cai Liu, Lin Wu, Xiao-Rong Zeng, Ji-Min Cao, Yan Wei

**Affiliations:** ^1^Department of Cardiology, The Affiliated Hospital of Southwest Medical University, Luzhou, China; ^2^Key Laboratory of Medical Electrophysiology of Ministry of Education and Medical Electrophysiological Key Laboratory of Sichuan Province, Collaborative Innovation Center for Prevention and Treatment of Cardiovascular Disease, Institute of Cardiovascular Research, Southwest Medical University, Luzhou, China; ^3^Department of Respiratory Medicine, Rongcheng People’s Hospital, Rongcheng, China; ^4^Department of Cardiology, Peking University First Hospital, Beijing, China; ^5^Key Laboratory of Cellular Physiology of Ministry of Education, Department of Physiology, Shanxi Medical University, Taiyuan, China

**Keywords:** sympathetic nerve, PC12 cell, differentiation, HCN channel, neurite outgrowth

## Abstract

Hyperpolarization-activated cyclic nucleotide-gated ion channels (HCN channels) are widely expressed in the central and peripheral nervous systems and organs, while their functions are not well elucidated especially in the sympathetic nerve. The present study aimed to investigate the roles of HCN channel isoforms in the differentiation of sympathetic neurons using PC12 cell as a model. PC12 cells derived from rat pheochromocytoma were cultured and induced by nerve growth factor (NGF) (25 ng/ml) to differentiate to sympathetic neuron-like cells. Sympathetic directional differentiation of PC12 cells were evaluated by expressions of growth-associated protein 43 (GAP-43) (a growth cone marker), tyrosine hydroxylase (TH) (a sympathetic neuron marker) and neurite outgrowth. Results show that the HCN channel isoforms (HCN1-4) were all expressed in PC12 cells; blocking HCN channels with ivabradine suppressed NGF-induced GAP-43 expression and neurite outgrowth; silencing the expression of HCN2 and HCN4 using silenced using small interfering RNAs (siRNA), rather than HCN1 and HCN3, restrained GAP-43 expression and neurite outgrowth, while overexpression of HCN2 and HCN4 channels with gene transfer promoted GAP-43 expression and neurite outgrowth. Patch clamp experiments show that PC12 cells exhibited resting potentials (RP) of about −65 to −70 mV, and also presented inward HCN channel currents and outward (K^+^) currents, but no inward voltage-gated Na^+^ current was induced; NGF did not significantly affect the RP but promoted the establishment of excitability as indicated by the increased ability to depolarize and repolarize in the evoked suspicious action potentials (AP). We conclude that HCN2 and HCN4 channel isoforms, but not HCN1 and HCN3, promote the differentiation of PC12 cells toward sympathetic neurons. NGF potentiates the establishment of excitability during PC12 cell differentiation.

## Introduction

The normal structure and function of sympathetic nerve is the key to maintain the normal physiological function of the heart (Gordan et al., 2015; Meng et al., 2018). A variety of heart diseases, including myocardial infarction (MI) and cardiomyopathy, can disrupt cardiac sympathetic innervation, in which sympathetic nerves are subjected to change both in structure and function in response to injury (Gardner et al., 2016). Unbalanced cardiac sympathetic innervation, including regional denervation and nerve sprouting/hyperinnervation, is strongly linked to the occurrence of arrhythmias in the course or prognosis of heart diseases (Cao et al., 2000a, b; Triposkiadis et al., 2009; Meng et al., 2018). The process of cardiac sympathetic nerve sprouting after injury includes axonal regeneration and outgrowth and neurilemmal cell proliferation (Fallavollita et al., 2014), this could ultimately lead to sympathetic nerve remodeling (Chen et al., 2001). In addition, postmortem studies have confirmed the evidence of sympathetic nerve sprouting in transplanted hearts, which showed that the sympathetic nerve density was more prominent in areas particularly around blood vessels, and the growth cone marker growth-associated protein 43 (GAP-43) and sympathetic nerve marker tyrosine hydroxylase (TH) were remarkably expressed in transplanted human hearts (Kim et al., 2004). Sympathetic nerve sprouting has both positive and negative effects, such as avoiding further injury by rerouting nerve signals around broken pathways and improving hemodynamic performance in the surviving myocardium, or contributing to the occurrence of malignant arrhythmias (Li and Li, 2015; Harrison et al., 2016). However, the underlying mechanisms of sympathetic nerve remodeling remains incompletely understood.

Besides the neural chemoattractant nerve growth factor (NGF) and the neural chemorepellent semaphorin 3a, several kinds of ion channels have been shown to play irreplaceable roles in sympathetic nerve development and remodeling. Among them, Na^+^ channels in mammalian peripheral axons take part in the process of nerve injury and neuroma formation (Devor et al., 1993). Knockdown of sodium channel Nav1.6 reduced sympathetic nerve sprouting (Xie et al., 2015). Cardiac sympathetic nerve sprouting inhibited the expressions and currents of myocardial *I*_to_ and *I*_K__1_ channels in rats (Ren et al., 2008), and also upregulated the expression of ionotropic glutamate receptors in rats with healed myocardial necrotic injury (Lu et al., 2012). In addition, non-voltage gated channels, such as transient receptor potential channels (TRPCs), regulate peripheral neuronal growth cones which are responsible for axon guidance and outgrowth (Shim and Ming, 2010). Furthermore, above mentioned and other studies on Ca^2+^, Na^+^, and K^+^ channels also showed powerful effects on growth cone responses to environmental changes during sympathetic remodeling (Gardner et al., 2016). However, the roles of hyperpolarization-activated cyclic nucleotide-gated ion channels (namely HCN channels) in controlling sympathetic nerve growth are largely unknown.

Hyperpolarization-activated cyclic nucleotide-gated ion channels show unique ion selectivity and can produce non-selective cationic currents through the cell membrane by permeating Na^+^ and K^+^ flux (Biel et al., 2009). HCN channel family comprises 4 isoforms (HCN1-4), which can be detected in heart and brain tissue, functioning for the *I*_h_ (or *I*_f_) current in regulating the rhythmic activity of cardiac pacemaker cells and spontaneously firing neurons (Benarroch, 2013; Novella Romanelli et al., 2016; Saponaro et al., 2019). During neuron and neural network development and maturation, HCN channels are involved in the neuronal responses to external stimuli by integrating a wide range of cellular signals (Bender and Baram, 2008). HCN channels are also localized at synapses and with high plasticity in function, maintaining intrinsic neuronal activity and synaptic transmission (Shah, 2014). Based on the current studies, HCN channels have wide important functions in neurological behavior, learning and memory, neuronal gain, central pattern generation, sensory perception, hormonal regulation, and cardiac pace making (Yang et al., 2015; Khurana, 2018). However, the action and mechanism of HCN channels in sympathetic neuronal differentiation and sprouting have seldom been studied.

The present study aimed to investigate the role of HCN channels in sympathetic nerve differentiation using PC12 cells which are derived from rat pheochromocytoma and have the ability of acquiring sympathetic neuron features in response to NGF (Hu et al., 2018; Terada et al., 2018), including neurite outgrowth (Peng et al., 2009) which is an important morphological feature and a key cellular process during neuronal differentiation (Vaudry et al., 2002). The study investigated the differential roles of HCN isoforms (HCN1-4) in NGF−induced PC12 cell differentiation. The expressions of TH and GAP-43 were used to identify the sympathetic nerve features of NGF-treated PC12 cells. The overall role of HCN channels in NGF-induced PC12 cell differentiation toward sympathetic neuron was checked by a pharmacological tool ivabradine (IVA), a non-selective blocker of HCN channels. The differential regulatory functions of each of the four HCN isoforms (HCN1-4) in PC12 cell differentiation were examined by overexpressing and silencing each of them. Results show that all the four HCN subchannels were expressed in PC12 cells. Blockade of HCN channels with IVA inhibited NGF−induced neurite outgrowth in PC12 cells. Overexpression of HCN2 or HCN4 by gene transfer intensified the sympathetic phenotype of PC12 cells as shown by increased expression of GAP-43; silencing HCN2 or HCN4 expression by siRNA weakened the sympathetic phenotype of PC12 cells as indicated by decreased expression of GAP-43; whereas HCN1 and HCN3 did not show significant effects on PC12 cell differentiation. The study suggests that HCN2 and HCN4 play important roles in PC12 cell differentiation toward sympathetic neuron.

## Materials and Methods

### Cell Culture

Rat pheochromocytoma (PC12) cell line was purchased from the National Infrastructure of Cell Line Resource (Beijing, China). Cells were cultured and passaged in 1640 medium (Gibco, United States) supplemented with 10% horse serum, 5% fetal bovine serum (Gibco, United States), penicillin (100 units/ml) and streptomycin (100 μg/ml) at 37°C in 5% CO_2_ atmosphere. To observe the neurite outgrowth, PC12 cells were seeded to 6 well plates at 5 × 10^4^ cells/well and cultured for 24 h. Then the culture medium was replaced by 1640 medium containing 10% HS, 5% FBS and NGF (25 ng/ml) with or without IVA (10 μmol/l). The change of neurite outgrowth in differentiated PC12 cells was defined as those with neurite length greater than the cell body of individual cell. The neurite outgrowth length was measured using NIS-Elements AR analyzer software.

### siRNAs, Plasmids, and Cell Transfection

Endogenous HCN1-4 expressions were siRNAs against each of the HCN1-4 isoforms. The siRNAs were chemically synthesized and purified by GenePharma Company (GenePharma, China). The sequences of these siRNAs were shown in Supplementary Table S1. To suppress HCN channel expression, PC12 cells were transfected with each of these synthesized siRNAs in the presence of Lipofectamine^®^ RNAiMAX reagent (Invitrogen, United States) following the instructions from the manufacturer. The pcDNA3.0 plasmids containing full-length HCN2 or HCN4 (pcDNA3.0/HCN2 or pcDNA3.0/HCN4) were kindly provided by Dr. Zuo from Southwest Medical University. To overexpress HCN channel, PC12 cells at 80–90% confluence were transiently transfected with 2.5 μg pcDNA3.0/HCN2 or pcDNA3.0/HCN4 plasmids using FuGENE^®^ HD Transfection Reagent (Roche, Germany) according to the manufacturer’s protocol. Forty-eight hours after transfection, cells were harvested for analyzing the expressions of HCN channels and related genes.

### Quantitative RT-PCR

Total RNA was extracted from cultured PC12 cells seeded in six well plate using trizol reagent (15596-018, Invitrogen), and reverse transcriptional reaction was conducted using moloney murine leukemia virus reverse transcriptase, random primers and oligo dT primers (RR047B, Takara). Quantitative PCR was performed by using CFX96 Real-Time PCR Detection System (Bio-Rad). The primers set across introns were designed to avoid genomic DNA contamination and were synthesized by Tsingke Co. (Chengdu, China). The PCR primer sequences were shown in Supplementary Table S2. Each reaction volume was 25 μl, which contained 1 × TB Green Premix Ex Taq II (Tli RNaseH Plus, Takara, Japan), 2 μl cDNA, 1 μl 400 nM forward primer and 1 μl 500 nM reverse primer. GAPDH was used as an internal standard for quantification. RNase-free water was used as a non-template control and non-reverse transcribed samples were run in parallel to exclude genomic DNA contamination. The real-time PCR cycle was programed with two steps. The initial step was 95°C for 30 s, then step two followed by 40 repeated cycles of 95°C for 5 s denaturation and 60°C annealing temperature for 30 s. The reactive conditions for real-time PCR were optimized by monitoring the melting temperature curve and by conducting the test of electrophoresis on agarose gels. The PCR products were confirmed by direct sequencing.

### Western Blotting

Cells were lysed with RIPA and the proteins were separated on 10% SDS-PAGE gel before transferring onto PVDF membranes. The membranes were blocked overnight with 5% BSA and then were respectively incubated with polyclonal anti-HCN1 (APC-056), anti-HCN2 (APC-030), anti-HCN3 (APC-057), anti-HCN4 (AGP-004) at 1:1000 dilution (Alomone Labs, Jerusalem, Israel), or with anti-GAP-43 (sc-135915) or anti-TH (sc-136100) at 1:1000 dilution (Santa Cruz Biotechnology, Inc. United States) overnight at 4°C. On the second day, blots were washed with phosphate buffered saline (PBS) and then incubated with a goat anti-rabbit IgG-HRP (sc-2004) or goat anti-mouse IgG-HRP (sc-2005) (1:2000 dilution) for 2 h at room temperature. Mouse anti-GAPDH (sc-32233) (1:2000 dilution) (Santa Cruz Biotechnology, Inc., United States) was used as an internal standard for protein quantification. After three washes with PBS, visualization was performed using enhanced chemiluminescence (ECL) plus the Western Blotting Detection system (Amersham Biosciences). Protein quantification was carried out with Image-Pro Plus (Media Cybernetics, Inc., United States) software.

### Immunofluorescent Staining

PC12 Cell expressions of HCN channels, GAP-43 and TH were detected using immunofluorescent staining. Cells were harvested by trypsinization, seeded on coverslips and incubated for 4 h before experiments. Cells were briefly washed with pre-warmed PBS at 37°C, and were fixed for 15 min in 4% paraformaldehyde with 0.12 mol/l sucrose in PBS (pH 7.4). After washing six times with PBS (5 min for each wash), cells were permeated by incubation in 0.5% Triton X-100 in PBS for 5 min, rinsed six times with PBS, blocked with 10% sheep serum in PBS for 45 min, washed once with 1% sheep serum in PBS, and incubated in a refrigerator (4°C) overnight in the presence or absence of the primary antibodies diluted with 1% sheep serum in PBS: 1:50 dilution for rabbit anti-HCN1 (APC-056), anti-HCN2 (APC-030), anti-HCN3 (APC-057), anti-HCN4 (AGP-004) (Alomone Labs, Jerusalem, Israel), anti-GAP43 (sc-135915) and anti-TH (sc-136100) (Santa Cruz Biotechnology, Inc., United States). Afterward, cells were washed six times with 1% sheep serum in PBS and incubated for 1 h in the secondary antibodies: Cy-3 conjugated goat anti-rabbit IgG for HCN isoforms, Alexa Fluor 594-conjugated goat anti-mouse IgG (R37121, Invitrogen, Carlsbad, CA) for GAP-43, and Alexa Fluor 594-conjugated goat anti-rabbit IgG (R37117, Invitrogen, Carlsbad, CA, United States) for TH (dilution 1:100 with 1% sheep serum in PBS). Finally, the coverslips with cells were washed six times in PBS, inverted onto mounting medium on glass slides and examined under a confocal microscope (C1-si, Nikon, Japan). The acquired images were analyzed using Nikon software (NIS-Elements AR, Nikon, Japan).

### Measurement of Neurite Outgrowth

The morphology of neurite outgrowth of cultured PC12 cells under different conditions were observed and calculated after culture for 3 days. Under a phase-contrast microscope, the lengths of neurite outgrowth were quantified based on more than 50 non-overlapping images (100×) of each group. The neurite was counted when it was longer than the diameter of the cell body. Total neurite length (sum of the lengths of all neurites extending from the cell body) and the maximal distance (length of longest neurite) were calculated. The average length of all the neurites in each group was calculated by dividing the total neurite length with cell numbers. The length of a neurite was automatically measured by the Nikon software (NIS-Elements AR, Nikon, Japan). Each experiment was conducted in triplicate. The expression of neuronal growth marker GAP-43 was also evaluated with immunofluorescent staining to indicate neuronal differentiation of PC12 cells.

### Patch Clamp to Measure Transmembrane Potentials and HCN and Other Channel Currents

Cultured PC12 cells treated or untreated with NGF were harvested by trypsinization, seeded on 6-mm coverslips coated with 10% poly-L-lysine solution 1 day before experiment. The coverslips with PC12 cells were mounted onto the recording chamber and visualized with infrared differential interference contrast optics (IX71, Olympus). The volume of the recording chamber was less than 1.0 ml and cells were perfused with recording solution at a flow rate of 4.0 ml/min. For recording transmembrane potentials, the recordings were performed at 37°C and current clamping mode was used with Axopatch 200B amplifier and Digidata 1555A (Molecular Devices). Signals were filtered at 10 kHz and sampled at 2 kHz using Clampex 10.6 software (Molecular Devices). Recording solution was composed of (in mmol/l) 140 NaCl, 5 KCl, 1.8 CaCl_2_, 1 MgCl_2_, 10 glucose (pH 7.4 adjusted with NaOH). Internal solution was composed of (in mmol/l) 110 K-aspartate, 20 KCl, 5 Na-phosphocreatine, 1 MgCl_2_, 5 ATP-2Mg, 0.1 GTP-2Na, 0.05 K2-EGTA, 10 HEPES (pH 7.2 adjusted with KOH). Pipettes were pulled with a pipette puller (P-97, Sutter Instrument Co.), pipette resistance was 3–5 MΩ when filled with standard internal solution. Liquid junction potential was 15 mV and was compensated. The recorded were analyzed with Clampfit 10.6 (Molecular Devices) and OriginPro 8.0 (Microcal Software Inc.). For HCN channel current recording, voltage clamp mode of whole cell configuration was used. The external solution was composed of (in mmol/l) 10 D-(+)-glucose, 5 HEPES, 110 NaCl, 25 KCl, 1.8 CaCl_2_, 1.2 MgCl_2_, 2 MnCl_2_, 0.2 CdCl_2_, 0.5 4-aminopyridine (pH 7.4 adjusted with NaOH); internal solution was composed of (in mmol/l) 130 K-L-aspartic-acid, 2 MgCl_2_, 5 ATP-2Na, 11 EGTA, 5 CaCl_2_, 10 HEPES (pH 7.2 adjusted with KOH). HCN currents were elicited by a use-dependence protocol, consisting of 11 consecutive hyperpolarizing pulse steps to −70 mV, followed by an I–V protocol, consisting of a series of hyperpolarizing steps to increase negative potentials from −40 to −140 mV from a holding potential of −40 mV. Baseline recording of HCN currents was stable for about 10 min. The steady-state amplitudes of HCN channel (Ih) currents at the end of each testing potential were measured and used to construct the I–V relationships. Current densities (pA/pF) were obtained by dividing the current amplitude with cell capacitance. Current densities at treatment conditions were plotted against respective clamping voltages to establish the I–V curves.

To elicit voltage-gated Na^+^ channel currents, holding potential was −90 mV. The voltage-dependent activation was generated by steps from −120 to 70 mV in 10 mV increments for 50 ms. To record outward (K^+^) currents, holding potential was −60 mV. The voltage-dependent activation was generated by steps from −60 to 60 mV in 10 mV increments for 400 ms.

### Statistical Analysis

Data were expressed as mean ± standard error (SEM). Student’s *t* test was used to compare the difference between the two groups. One-way ANOVA was used for multiple groups comparison, and Bonferroni test was used to compare between the two groups. A significant level of *p* < 0.05 was used for all comparisons; R 3.5.1 software was used for statistical analysis.

## Results

### NGF Induces PC12 Cell Differentiation Toward Sympathetic Neurons

**FIGURE 1:**
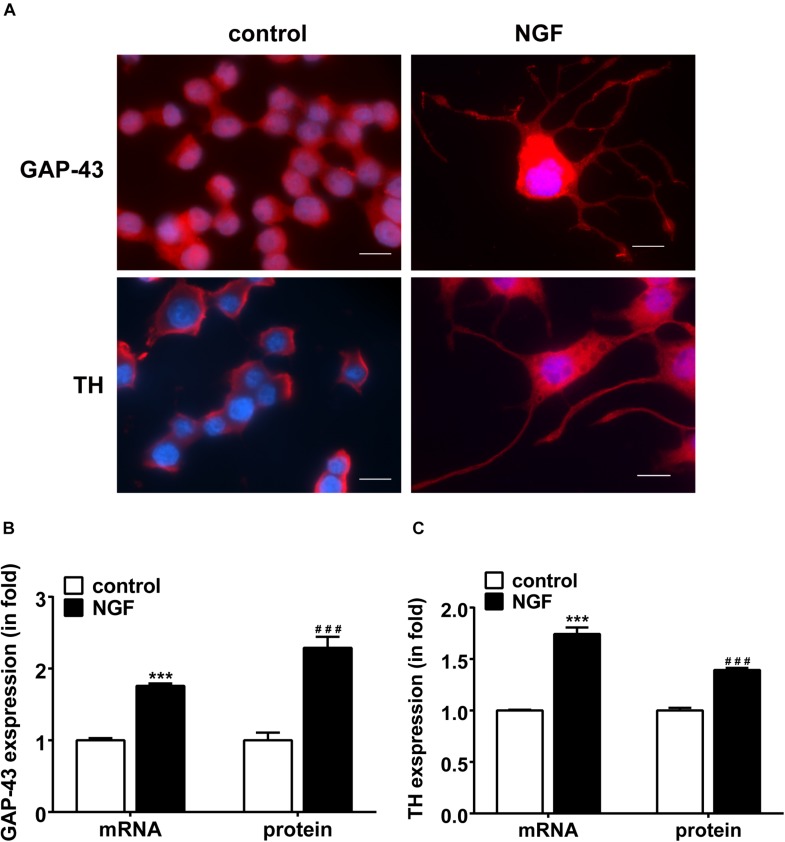
NGF-induced PC12 cell differentiation toward sympathetic neurons and expressions of GAP-43 and TH. **(A)** Immunofluorescent stains of GAP-43 (red, upper row) and TH (red, lower row). Nuclei were stained blue by DAPI. Note that NGF induced significant neurite outgrowth. Scale bar, 10 μm. **(B,C)** Real-time PCR and western blotting results of GAP-43 and TH expressions in PC12 cells treated or untreated with NGF. ^∗∗∗^*p* < 0.001 vs. respective control. ^###^*p* < 0.001 vs. respective control; *n* = 3 for each value.

Immunofluorescent staining was used to examine the neuronal outgrowth marker GAP-43 and sympathetic neuronal marker TH in PC12 cells. Figure 1A displays the NGF-induced morphological change of PC12 cells. Under the induction of NGF, the morphology of PC12 cells changed from an original round shape to a shape with neurite outgrowth. Figures 1B,C shows that NGF increased the expression of GAP-43 and TH in PC12 cells along with elongation of neurite outgrowth, suggesting differentiation toward sympathetic neurons.

### HCN Channels Are Expressed in PC12 Cells

**FIGURE 2:**
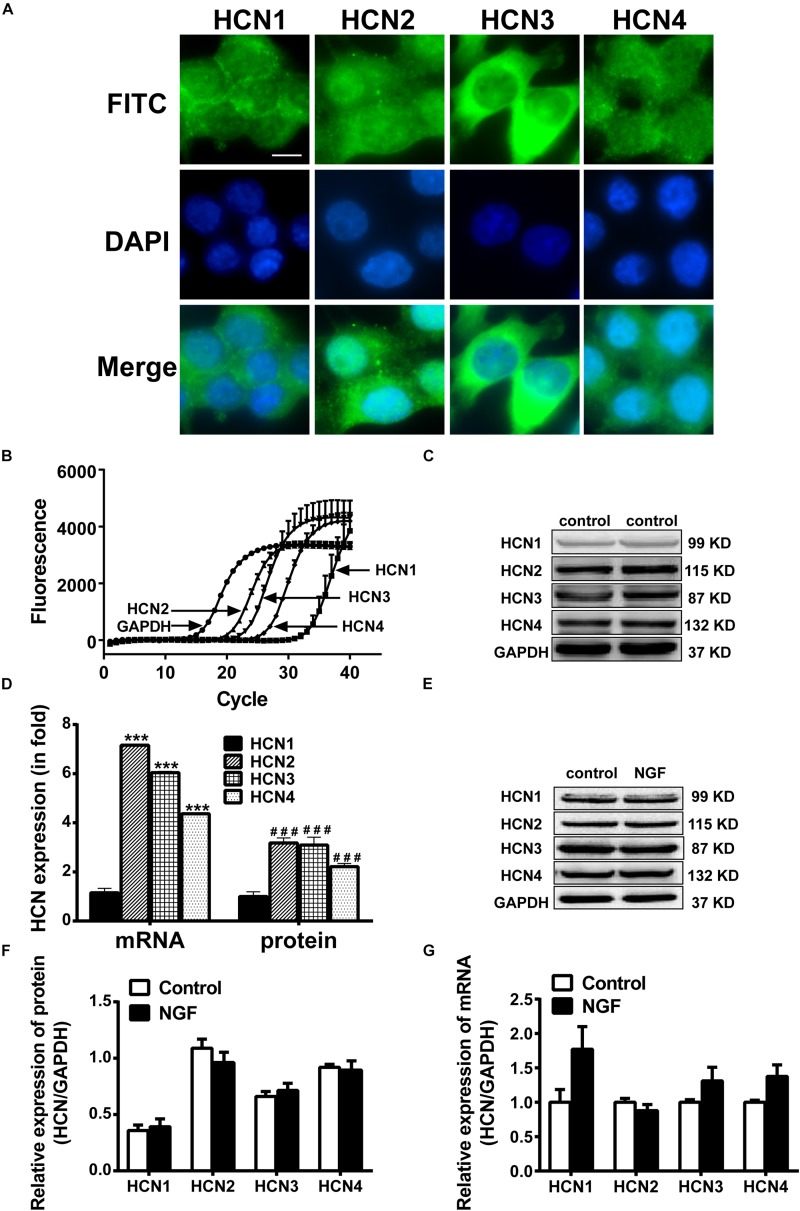
Expressions of HCN channel isoforms in PC12 cells with or without NGF treatment. **(A)** Immunofluorescent stains of HCN1-4 proteins (green) in PC12 cells without NGF treatment. Scale bar, 10 μm for all subpanels. DAPI was used for cell nuclei staining (blue). **(B)** Representative real-time PCR amplification plots for HCN isoforms and GAPDH in PC12 cells without NGF treatment (cycle numbers vs. fluorescence). **(C)** Representative western blot electrophoresis bands in PC12 cells without NGF treatment. **(D)** Statistical results of real-time PCR and western blots of HCN isoforms in NGF-untreated PC12 cells. ^∗∗∗^*p* < 0.001 vs. the mRNA level of HCN1. ^###^*p* < 0.001 vs. the protein level of HCN1. **(E)** Representative western blots of HCN isoforms in NGF-treated and NGF-untreated PC12 cells. **(F)** Statistical results of protein levels for HCN channel isoforms in NGF-treated and NGF-untreated PC12 cells. **(G)** Statistical results of mRNA levels for HCN channel isoforms in NGF-treated and NGF-untreated PC12 cells.

Real-time PCR, western blotting and immunofluorescency were employed to detect the mRNA and protein expressions of HCN channel isoforms in NGF-treated PC12 cells. Immunofluorescence results (Figure 2A) clearly reveal that the positive signals (green fluorescence) representing HCN1-4 channels mainly localized on the membranes. Real-time PCR results (Figures 2B,C) show that the mRNA level of HCN1 was very low in PC12 cells, while the mRNA levels of HCN2, HCN3, and HCN4 were relatively higher compared with the reference gene GAPDH. The protein expression levels of HCN1-4 isoforms were respectively consistent with their mRNA levels (Figures 2C,D). In short, the HCN channel accumulations (median values) in PC12 cells, based on the results of real-time PCR and western blotting, were HCN2 > HCN3 > HCN4 > HCN1. Besides, NGF treatment did not significantly affect the mRNA and protein expression levels of HCN1-4 isoforms in PC12 cells (Figures 2E–G).

### Blockade of HCN Channels Inhibits Neurite Outgrowth in PC12 Cells

**FIGURE 3:**
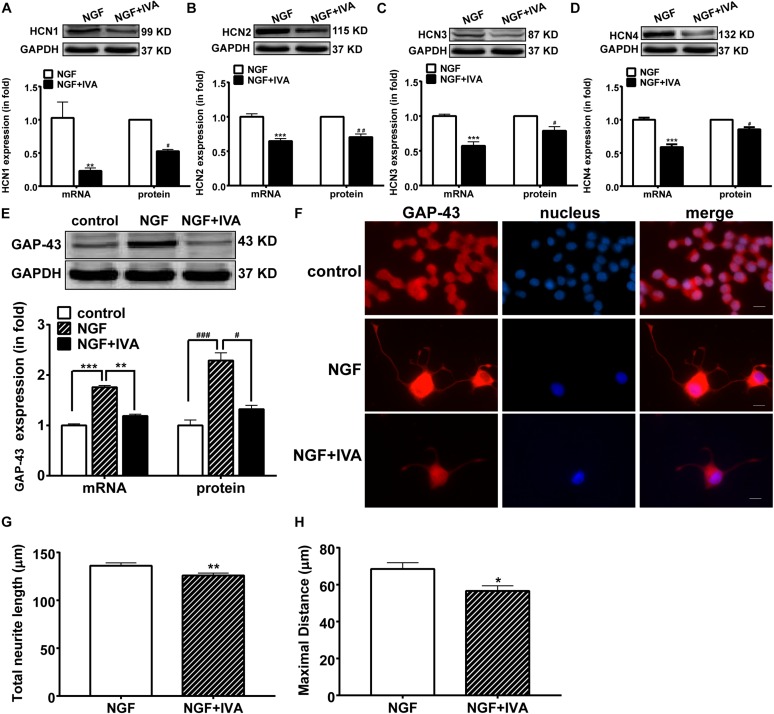
The suppressing effects of ivabradine (IVA) on the mRNA and protein expressions of HCN channel isoforms and GAP-43 and the neurite outgrowth. **(A–D)** Real-time PCR and western blotting results of the mRNA and protein expression levels respectively for HCN1-4 isoforms in NGF-treated PC12 cells relative to GADPH level. ^∗∗^*p* < 0.01, ^∗∗∗^*p* < 0.001 vs. NGF treatment alone in the mRNA level. ^#^*p* < 0.05, ^##^*p* < 0.01 vs. NGF treatment alone in the protein level. **(E)** Representative western blotting bands (*upper*) and the mRNA and protein semiquantitative values (*lower*) of GAP-43 in NGF-treated PC12 cells. ^∗∗^*p* < 0.01, ^∗∗∗^*p* < 0.001 vs. NGF treatment alone in the mRNA level. ^#^*p* < 0.05, ^###^*p* < 0.001 vs. NGF treatment alone in the protein level. **(F)** Immunofluorescent stains of GAP-43 protein (red), displaying that NGF induced, while ivabradine inhibited, the neurite outgrowth in differentiating PC12 cells. DAPI was used for cell nuclei staining (blue). Scale bar, 10 μm. **(G,H)** Quantitative morphological parameters of neurite outgrowth including the total neurite length and the maximal neurite length (longest process per cell). Results are calculated from three independent experiments and presented as mean ± SEM, 96 < *n* < 213, ^∗^*p* < 0.05, ^∗∗^*p* < 0.01 vs. NGF treatment alone.

To further identify whether HCN channels regulate neurite outgrowth, we observed the effect of IVA (10 μmol/l) on HCN channels, GAP-43 expression and NGF-induced neurite outgrowth in PC 12 cells. Real-time PCR and western blotting results showed that IVA treatment for 72 h decreased the expressions of HCN isoforms in PC12 cells treated with NGF plus IVA: HCN1 expression was decreased by 77.57 ± 4.20% (*p* < 0.01, *n* = 7) in the mRNA level and decreased by 57.97 ± 3.00% (*p* < 0.05, *n* = 7) in the protein level; HCN2 expression was decreased by 35.30 ± 3.56% (*p* < 0.001, *n* = 7) in the mRNA level and decreased by 29.50 ± 4.53% (*p* < 0.01, *n* = 3) in the protein level; HCN3 expression was decreased by 42.90 ± 5.80% (*p* < 0.001, *n* = 6) in the mRNA level and decreased by 21.22 ± 5.98% (*p* < 0.05, *n* = 3) in the protein level; HCN4 expression was decreased by 41.07 ± 3.80% (*p* < 0.001, *n* = 9) in the mRNA level and decreased by 12.23 ± 2.93% (*p* < 0.05, *n* = 5) in the protein level (Figures 3A–D). These results indicate that blocking HCN channels with IVA inhibited the expressions of all the four HCN channel isoforms both in the mRNA and protein levels.

We then examined the effect of IVA on the expression of GAP-43. In normal culture medium, NGF significantly increased the mRNA and protein expressions of GAP-43 in PC12 cells compared with the control group, whereas IVA suppressed the increase of GAP-43 expression induced by NGF (Figure 3E).

As IVA affected the expression of GAP-43 in differentiating PC12 cells, we also checked the effects of IVA on the differentiation and neurite length of PC12 cells. The total neurite length and maximal neurite length were measured. Three days after NGF treatment with or without IVA, neuronal differentiation and neurites in PC12 cells were observed by staining GAP-43 and neurite length. PC12 cells treated with NGF plus IVA showed shorter neurites than cells treated with NGF alone (Figure 3F). To statistically evaluate the effect of IVA on neurites outgrowth, we counted the lengths of neurites, the average total neurite length in cells with NGF treatment alone was 136.10 ± 3.00 μm (*n* = 172) and the maximal neurite length was 68.49 ± 3.38 μm (*n* = 154) (*p* < 0.05) (Figures 3G,H); while in cells treated with NGF plus IVA, the total neurite length was 126.00 ± 2.43 μm (*n* = 213) and the maximal neurite length was 56.58 ± 2.83 μm (*n* = 96) (*p* < 0.05) (Figures 3G,H), indicating that IVA hampered the neurite outgrowth.

### Knocking-Down HCN2 and HCN4 Inhibits GAP-43 Expression and Neurite Outgrowth in PC12 Cells

**FIGURE 4:**
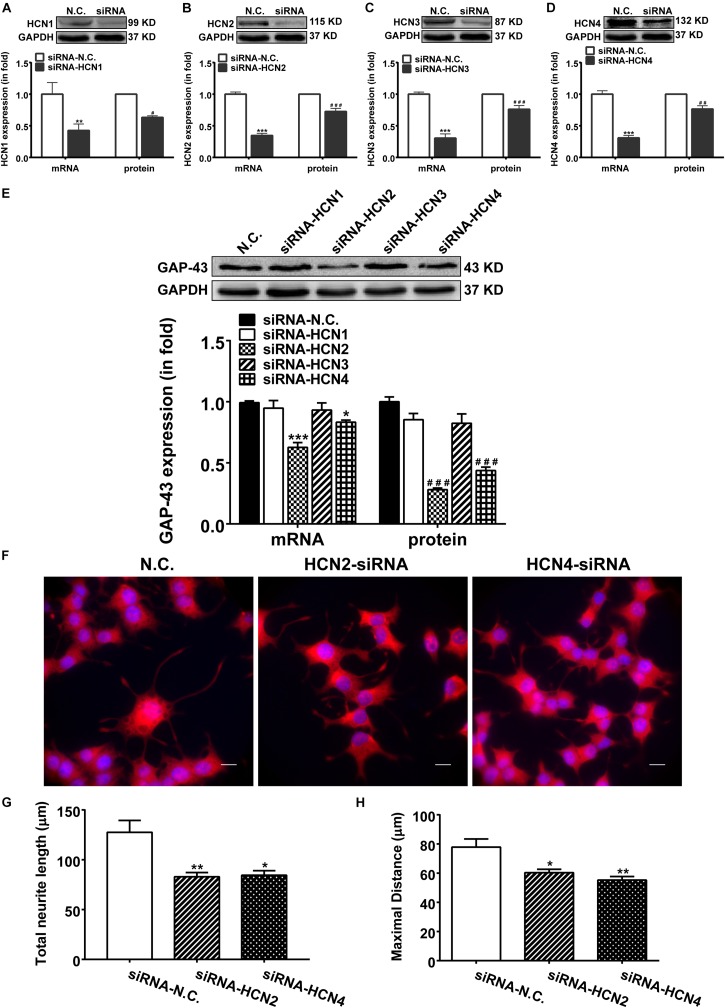
Knocking-down HCN2 and HCN4 expression with siRNAs inhibited GAP-43 expression and neurite outgrowth in PC12 cells. NGF-treated PC12 cells were transiently transfected with the negative control siRNA (NC-siRNA) or with the siRNA respectively for HCN1, HCN2, HCN3 or HCN4 using Lipofectamine^®^ RNAiMAX Reagent for 48 h before cells were harvested for detection. **(A–D)** The mRNA and protein expressions of HCN1-4 isoforms assayed by qPCR and western blotting in comparison to GADPH in NGF-treated PC12 cells. ^∗∗^*p* < 0.01, ^∗∗∗^*p* < 0.001 vs. negative control siRNA (N.C.) in the mRNA level. ^#^*p* < 0.05, ^##^*p* < 0.01, ^###^*p* < 0.001 vs. N.C. in the protein level. **(E)** Representative western blotting results (*upper*) and the densitometric analysis of GAP-43 mRNA and protein expressions (*lower*) (*n* = 3–6 independent experiments). ^∗^*p* < 0.05, ^∗∗∗^*p* < 0.001 vs. N.C. in the mRNA level. ^###^*p* < 0.001 vs. N.C. in the protein level. **(F)** Immunofluorescent stains of GAP-43 in PC12 cells transiently transfected with siRNA targeting HCN2 or HCN4. Scale bar, 10 μm. **(G,H)** Quantification of neurite morphological parameters (total length of neurite outgrowth and longest process per cell). Results are calculated from three independent experiments and presented as mean ± SEM, 50 < *n* < 84, ^∗^*p* < 0.05, ^∗∗^*p* < 0.01 vs. N.C.

Above results indicate that blocking HCN channels with IVA exerted inhibitory effects on GAP-43 expression, differentiation and neurite outgrowth in PC12 cells. We further investigated which subtype of HCN channels was in responsible for the phenomenon. We used siRNA technique to specifically knockdown the expression of each HCN isoform. Respective transfection with siRNA-HCN1-4 decreased both the mRNA and protein levels of the respective HCN1-4 isoform in PC12 cells compared with the negative control PC12 cells (Figures 4A–D). Knocking-down HCN2 and HCN4 accordingly decreased both the mRNA and protein expressions of GAP-43 in PC12 cells (Figure 4E). The mRNA levels of GAP-43 were decreased by 38.75 ± 3.63% (*p* < 0.001, *n* = 17) and 17.25 ± 1.21% (*p* < 0.05, *n* = 17), respectively by knocking-down HCN2 and HCN4 (Figure 4E, left), the protein levels of GAP-43 were decreased by 60.60 ± 5.32% (*p* < 0.001, *n* = 6) and 55.82 ± 3.60% (*p* < 0.001, *n* = 6), respectively by knocking-down HCN2 and HCN4 (Figure 4E, right), compared with the negative control PC12 cells. While knocking-down HCN1 and HCN3 did not significantly affect the mRNA and protein expressions of GAP-43 (Figure 4E).

More important and immediate intuitive finding in this study was that silencing either HCN2 or HCN4 channel with siRNA technique both decreased the outgrowth of PC12 cells (Figure 4F). Cells transiently transfected with siRNA targeting HCN2 or HCN4 revealed significantly shorter neurites, the total neurite length was 82.95 ± 14.19 μm (*p* < 0.01, *n* = 75) and the maximal neurite length was 60.30 ± 6.32 μm (*p* < 0.05, *n* = 84) in cells knocking down HCN2; the total neurite length was 84.45 ± 14.35 μm (*p* < 0.05, *n* = 71) and the maximal neurite length was 55.23 ± 6.56 μm (*p* < 0.01, *n* = 70) in cells knocking down HCN4 (Figures 4G,H), compared respectively with the negative control PC12 cells which showed total neurite length of 127.60 ± 11.88 μm (*n* = 50) and maximal neurite length of 77.83 ± 5.61 μm (*n* = 50) (Figures 4G,H).

### Overexpression of HCN2 and HCN4 Enhances GAP-43 Expression and Neurite Outgrowth in PC12 Cells

**FIGURE 5:**
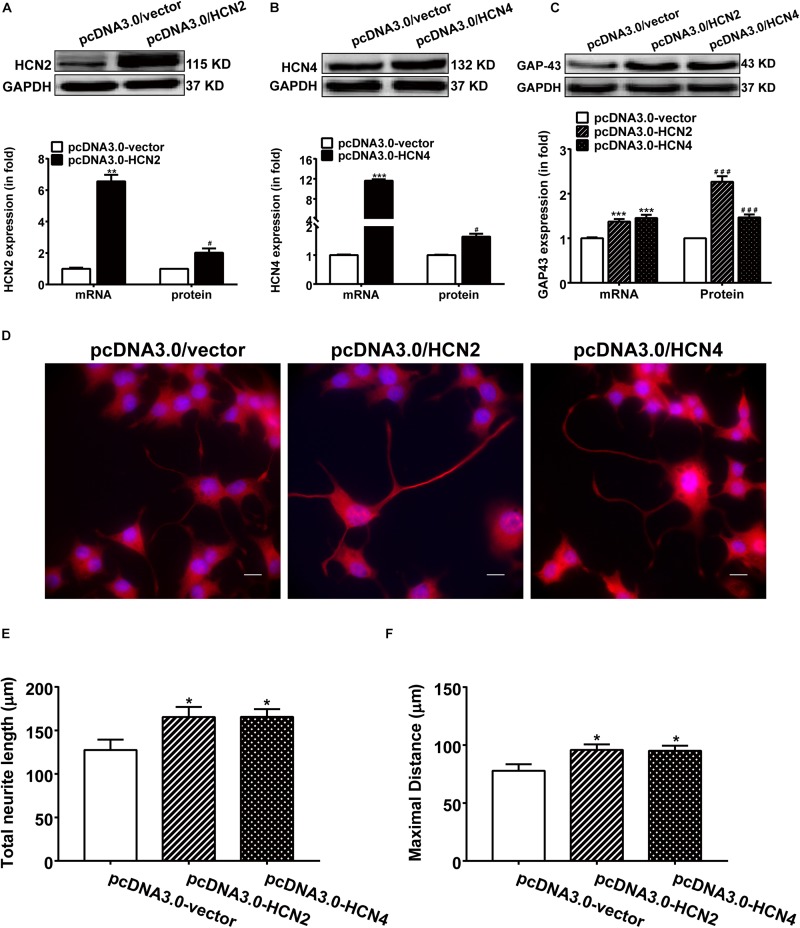
Overexpression of HCN2 and HCN4 enhanced GAP-43 expression and neurite outgrowth in PC12 cells. Results were obtained 48 h after cell transfection with either empty plasmid pcDNA3.0/vector or pcDNA3.0/HCN2 or pcDNA3.0/HCN4. **(A,B)** Representative western blots (*upper*) and semiquantitative values (*lower*) of the mRNA and protein levels of HCN2 and HCN4 isoforms. ^∗∗^*p* < 0.01, ^∗∗∗^*p* < 0.001 vs. control (pcDNA3.0/vector) in the mRNA level. ^#^*p* < 0.05 vs. control (pcDNA3.0/vector) in the protein level, *n* = 4−6 independent experiments. **(C)** qPCR and western blotting results showing the effects of HCN2 or HCN4 overexpression on the mRNA and protein expressions of GAP-43. ^∗∗∗^*p* < 0.001 vs. empty plasmid in the mRNA level. ^###^*p* < 0.001 vs. empty plasmid in the protein level, *n* = 4−6 independent experiments. **(D)** Immunofluorescent stains of GAP-43 (red) of PC12 cells transiently transfected respectively with pcDNA3.0/vector, pcDNA3.0/HCN2 or pcDNA3.0/HCN4 plasmids. Scale bar, 10 μm. **(E,F)** Quantification of neurite morphological parameters including total neurite outgrowth and longest process per cell. Results are calculated from three independent experiments and presented as mean ± SEM, 50 < *n* < 90, ^∗^*p* < 0.05 vs. empty vector.

Overexpression of HCN2 and HCN4 were achieved respectively by transfecting PC12 cells with pcDNA3/HCN2 and pcDNA3/HCN4 plasmids. Transfection with either HCN2 or HCN4 plasmids remarkably increased the mRNA and protein levels of HCN2 and HCN4 in PC12 cells (Figures 5A,B). Specifically, transfection with pcDNA3/HCN2 plasmid increased the GAP-43 mRNA level by 555.69 ± 41.96% (*p* < 0.001, *n* = 6) and increased the GAP-43 protein level by 126.80 ± 12.34% (*p* < 0.05, *n* = 4); transfection with pcDNA3/HCN4 plasmid increased the GAP-43 mRNA level by 45.95 ± 6.93% (*p* < 0.001, *n* = 6) and increased the GAP-43 protein level by 36.65 ± 10.13% (*p* < 0.01, *n* = 6) (Figure 5C). It is obvious that HCN2 was more powerful in stimulating GAP-43 protein expression than HCN4.

As expected, contrary to the silencing experiments, over expression of HCN2 or HCN4 channels increased the neurite outgrowth in NGF-treated PC12 cells (Figure 5D). Cells transiently transfected with pcDNA3.0/HCN2 or pcDNA3.0/HCN4 plasmids revealed significantly longer neurites: cells overexpressing HCN2 showed a total neurite length of 165.40 ± 13.71 μm (*p* < 0.05, *n* = 90) and a maximal neurite length of 95.82 ± 6.30 μm (*p* < 0.05, *n* = 86); cells overexpressing HCN4 yielded a total neurite length of 164.5 ± 13.83 μm (*p* < 0.05, *n* = 85) and a maximal neurite length of 95.03 ± 6.32 μm (*p* < 0.05, *n* = 84) (Figures 5E,F), compared to control PC12 cells transfected with empty vector in which the total neurite length was 127.57 ± 11.88 μm (*n* = 53) and the maximal neurite length was 77.83 ± 5.61 μm (*n* = 50). These results indicate that upregulating the expression of HCN2 or HCN4 enhanced NGF-induced PC12 cell differentiation neurite outgrowth.

### Phenotypes of NGF-Untreated and NGF-Treated PC12 Cells in the Resting Membrane Potential, Excitability, and HCN Channel Currents

**FIGURE 6:**
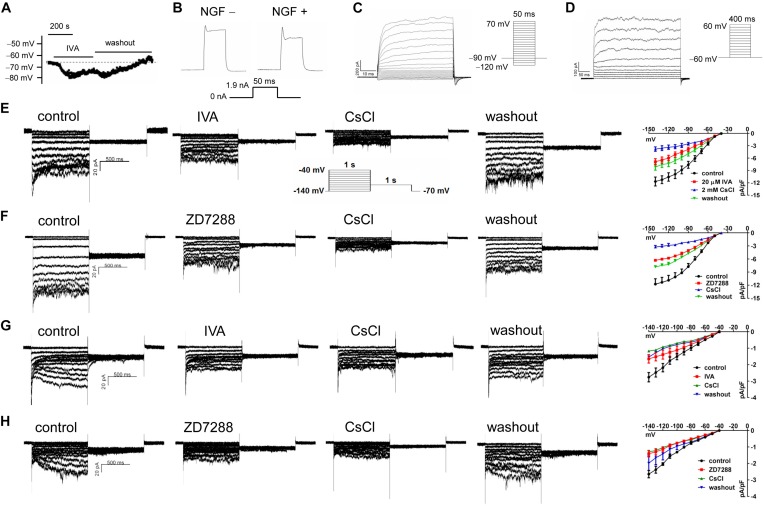
The transmembrane potentials and channel currents of PC12 cells with or without NGF treatment. **(A)** Resting potential (RP) recorded at a current-clamping mode in NGF-pretreated PC12 cells, note that blocking HCN channels with IVA (10 μmol/l) hyperpolarized the RP. **(B)** Suspicious action potentials evoked by electric pulses in NGF-untreated and NGF-treated PC12 cells (recorded at a current-clamping mode). Note that cells showed regular depolarization but immature repolarization potentially because of the large outward rectification. NGF treatment likely improved the repolarization. **(C)** Macrocurrents elicited by a stimulating protocol for activating voltage-gated Na^+^ channel in the absence of any channel blocker. No inward (supposing Na^+^) current was recorded, suggesting that PC12 cells did not develop mature voltage-gated Na^+^ channel even treated with NGF for certain time. **(D)** Macrocurrents recorded using a stimulating protocol for activating K^+^ channels in the absence of any channel blocker. Outward (K^+^) currents were recorded. **(E,F)** HCN channel currents recorded in NGF-untreated PC12 cells at a voltage clamping mode. The currents were blocked or partially blocked by IVA **(E)**, CsCl **(E,F)**, and ZD7288 **(F)**. **(G,H)** HCN currents recorded in NGF-treated PC12 cells. Note that the HCN currents were obviously smaller than that in NGF-untreated PC12 cells. Again, the currents were blocked or partially blocked by IVA, CsCl and ZD7288. The far right subpanels in panels **(E–H)** are the respective I–V curves which reflect the HCN current densities at different clamping voltage.

The resting potential (RP) of PC12 cells were recorded using a current clamping mode of patch clamp. Figure 6A shows a representative RP of about −65 mV in NGF-treated PC12 cells at baseline, and the RP and was hyperpolarized by IVA and recovered after washout. Statistical analysis shows no significant difference in the RPs between NGF-untreated PC12 cells (−69.82 ± 1.03 mV) and NGF-treated PC12 cells (−64.73 ± 1.67 mV). In the presence of IVA, the RP was hyperpolarized to −71.28 ± 1.91 mV in NGF-treated PC12 cells (*p* < 0.05 vs. NGF-treated PC12 cells at baseline). Suspicious action potential (AP) was elicited by certain depolarizing pulses in PC12 cells (Figure 6B), but there was strong delayed rectification in the “repolarizing” phase, suggesting that PC12 cells especially those treated with NGF may possess certain ability to depolarize and potentially be able to generate AP, but the ability is weak and thus they are not typical excitable cells even treated with NGF for certain times.

To determine whether voltage-gated Na^+^ channels were present in the PC12 cells, we used a stimulating protocol to activate voltage-gated Na^+^ channel in the absence of any channel blocker. Inward (supposing Na^+^) current could not be elicited by this approach either in NGF-untreated PC12 cells or NGF-treated PC12 cells (Figure 6C). Recording of macrocurrents using a stimulating protocol for activating K^+^ channels in the absence of any channel blocker shows that outward (supposing K^+^) currents could be elicited in PC12 cells treated with or without NGF (Figure 6D).

HCN channel currents were recorded in both NGF-untreated and NGF-treated PC12 cells using a voltage clamp mode (Figures 6E–H). The HCN currents were confirmed by applying CsCl (a non-selective cation channel blocker), IVA (a non-selective HCN channel blocker), and ZD7288 (an alternative HCN channel blocker) to the cell perfusion dish. The I–V curves of HCN channels (shown in the far right subpanels of Figures 6E–H) indicate HCN current densities under different conditions. For example, CsCl at 2 mmol/l strongly reduced the peak HCN current density from −11.68 ± 1.09 pA/pF to −3.80 ± 0.55 pA/pF at −140 mV (*p* < 0.01 vs. control, *n* = 8); IVA at 10 μmol/l decreased the peak HCN current density from −11.68 ± 1.09 pA/pF to −6.89 ± 0.76 pA/pF at −140 mV voltage in the NGF-untreated PC12 cells (*p* < 0.05 vs. control, *n* = 6); ZD7288 at 10 μmol/l reduced the peak HCN current density from 11.77 ± 1.24 pA/pF to −6.30 ± 0.28 pA/pF at −140 mV voltage (*p* < 0.05 vs. control, *n* = 5). Note that the HCN channel currents were smaller in NGF-treated PC12 cells than cells untreated with NGF (Figures 6G,H vs. Figures 6E,F), suggesting that NGF mildly inhibits HCN channels of PC12 cells at least in the functional aspect.

## Discussion

We demonstrated here that all the four HCN channel isoforms (HCN1-4) were expressed in PC12 cells and NGF promoted PC12 cell differentiation potentially by affecting the expression and function of certain HCN channels. For example, blockade of HCN channels inhibited the neurite outgrowth in PC12 cells; knocking-down HCN2 or HCN4 channel inhibited GAP-43 expression and neurite outgrowth, while overexpression of HCN2 or HCN4 enhances enhanced GAP-43 expression and neurite outgrowth in PC12 cells. These results suggest that HCN2 and HCN4 channels differentially take part in the differentiation of PC12 cells toward sympathetic neurons.

PC12 cells differentiation to sympathetic neuron-like cells by NGF was featured by expressions of TH and GAP-43, which are considered the indicators of neurite outgrowth and synaptogenesis (Das et al., 2004; Holahan, 2015; Huang et al., 2015). We observed the typical growth cone-like extensions and the morphological changes of PC12 cells from a round shape to a shape with neurite outgrowth (shown in Figure 1A), and the expression of TH and GAP43 was upregulated along with the elongation of neurite outgrowth induced by NGF in PC12 cells (shown in Figure 1B). GAP43, a growth-associated protein, plays an important role in the developmental regulation of axonal growth and neural network formation (Holahan, 2015), and its upregulation in the levels of mRNA and protein is also necessary and decisive for the differentiation of PC12 cells (Jap Tjoen San et al., 1991, 1995). TH, a rate-limiting enzyme in catecholamine biosynthesis, can reflect the activity of dopaminergic/adrenergic neurons and participate in neuronal plasticity (Jia et al., 2015). Therefore, NGF-induced differentiation of PC12 cells suggests a differentiation toward sympathetic neurons.

The HCN channel isoforms are widely expressed in the central nervous system and peripheral tissues and nerves, contributing to neuronal function and cardiac rhythmicity (Shah, 2014), while the functions of HCN channels in PC12 cells and their roles in NGF-induced differentiation have not been well studied. We found that the four HCN isoforms existed in NGF-induced PC12 cells, quantitatively defined as: HCN2 > HCN3 > HCN4 > HCN1 (shown in Figure 2). In the central nervous system, HCN1 is mainly expressed in the neocortex, hippocampus, brainstem, spinal cord, dorsal root ganglion and cerebellar cortex; HCN2 highly exists in thalamus, brainstem nuclei, external of the globuspallidus (GPe), small nociceptive DRG neurons and almost all brain regions; HCN3 can be detected in the olfactory bulb and hypothalamus, but little is known about its function because of low levels in the CNS; HCN4 is extensively distributed in thalamic nuclei, basal ganglia, habenular complex and the olfactory bulb (Herrmann et al., 2007; Benarroch, 2013; He et al., 2014; Kim and Johnston, 2018). In addition, HCN isoforms (HCN1-4) can be identified in different cardiac regions, such as sinoatrial node, atrioventricular node, purkinje fibers, ventricle and atrium, with the principal properties of native cardiac *I*_h_ (Biel et al., 2002). More important to the sympathetic nervous system, Kullmann et al. (2016) detected the mRNAs of HCN1-4 in rat superior cervical ganglion (SCG), although they did not find the protein expressions of these four isoforms; Tallapragada et al. (2016) reported that cAMP–dependent HCN channels were related to sympathetic nerve activity in the ventrolateral medulla of Sprague-Dawley rats, suggesting that HCN channels may also regulate sympathetic activity at the central site. Similarly, our present results about HCN channels expression in PC12 cells suggest that NGF-induced PC12 cell differentiation can be used as a model to study the relationship among HCN isoforms, sympathetic neuronal behavior and sympathetic nerve sprouting.

Neurite outgrowth occurs in the developing neurons and during nerve regeneration (Richardson and Shen, 2019), providing a visual representation of neuronal behaviors induced by particular substrates or exogenous factors (Filous and Silver, 2016). Variation of neurite outgrowth *in vitro* can be measured and expressed as total neurite length and maximal distance (Shepard et al., 2012). The PC12 cell is a well-established model system which can be used for investigating neuronal differentiation and function through various interventions (Kudo et al., 2015; Lynch et al., 2018). In the present study, pharmacological tool (IVA) and molecular interference (gene silence or overexpression) were used in PC12 cells to disturb HCN channels. These strategies led to changes in GAP-43 and neurite outgrowth, confirming the effects of HCN channels on PC12 cell differentiation. Similar to induced differentiation of PC12 cells via other factors, such as temperature-controlled repeated thermal stimulation (Kudo et al., 2015), crosslinked silica aerogels (Lynch et al., 2018), and gelsolin overexpression (Furnish et al., 2001), our results show the same morphology change of neurite outgrowth as in these studies. Alternative studies have shown that the expressions of functional voltage-sensitive K^+^ and Na^+^ channels have a profound influence on PC12 cell differentiation, resulting in neurite outgrowth and morphological change (Mandel et al., 1988; Sharma et al., 1993). However, there is still no report to show the regulatory role of HCN channels on neuronal differentiation and neurite outgrowth in PC12 cells, although elaborated reviews have summarized the HCN channels in developing neuronal networks and the influences of K^+^ and Ca^2+^ channels on the role of HCN channels (Bender and Baram, 2008; Kase and Imoto, 2012; Shah, 2014). Here, we show that the mRNA and protein levels of HCN channels were regulated by NGF (Figures 2E–G), and were decreased by HCN channel blocker IVA and by siRNA in NGF-treated PC12 cells. Importantly, GAP-43 and neurite outgrowth was reduced when HCN channels were blocked or knocked down, indicating the involvement of HCN channels in PC12 cell differentiation. By contrast, overexpression of HCN channels increased the GAP-43 level and the neurite outgrowth, confirming the regulatory role of HCN channels on PC12 cell differentiation.

Clinical study and fundamental research suggest that IVA reverses electrical and mechanical remodeling, adjusting the imbalance of the autonomic nervous system with sympathetic activation (Milliez et al., 2009; Kucerova et al., 2018). We show that IVA inhibited HCN channel expression and current density, these results are in consistent with previous reports (Suffredini et al., 2012; Li et al., 2015). IVA also inhibited NGF-induced neurite outgrowth in PC12 cells, this finding provides us with an in-depth understanding on the function of HCN channels in sympathetic nerve modeling. Alternative studies have shown that IVA inhibits the expressions of HCN1, HCN2 and HCN4 in HEK293 cells (Postea and Biel, 2011), and plays a role in human HCN4 as an “open−channel” blocker and as a “closed−channel” blocker in mouse HCN1 (Bucchi et al., 2006). Our results indicated that blocking HCN channels by IVA inhibited the expressions of HCN1–4 both in the mRNA and the protein levels, proving again that IVA is a non-selective blocker for HCN channels but not a selective blocker for any of the four HCN channel isoforms (Novella Romanelli et al., 2016). These studies could comprehensively explain the effect of IVA on the differentiation and neurite outgrowth of PC12 cells.

Changes in HCN channel phenotype due to gene transcriptional imbalance may disrupt neuronal network activity and contribute to disease pathogenesis. It is still unclear how the change of HCN channel phenotype happens in sympathetic neurons and how the change would impact on organs innervated with sympathetic nerves in diseased states. Therefore, deep exploration on the functions of HCN channels in GAP-43 expression and neurite outgrowth may contribute to unlocking the neurologic mechanisms of sympathetic nerve sprouting in cardiac diseases such as MI. An interesting finding of the present study was that HCN channel isoforms exerted differential regulatory roles in PC12 cell differentiation toward sympathetic neurons: HCN2 and HCN4, but not HCN1 and HCN3, exerted significant roles in regulating GAP-43 expression and neurite outgrowth in NGF-treated PC12 cells. Researchers have reported the action of cAMP on HCN channels based on analyzing between a series of deletion mutants and HCN1/HCN2 chimeras (Wainger et al., 2001; Wang et al., 2001), implying that HCN isoform co-assembles are special features in organisms. Immunocytochemical studies on CHO cells and HEK-293 cells showed that HCN2-HCN4 heteromeric channels co-assemble to form functional heteromeric channels which had a colocalization and a positive correlation with the embryonic mouse heart development (Whitaker et al., 2007; Ye and Nerbonne, 2009). Similar to our results, HCN2 and HCN4 simultaneously changed in regulating GAP-43 in PC12 cells, further verifying the opinion that functional co-assembly of HCN2 and HCN4 is important in native tissue (Whitaker et al., 2007; Ye and Nerbonne, 2009), although co-immunoprecipitation experiment was not conducted in the study. Furthermore, it has been demonstrated that mouse and human HCN2 and HCN4 channels underwent mode shifts by displaying changes in their tail currents (Elinder et al., 2006). But a recent study suggests that there is no evidence that HCN2 and HCN4 can form the heteromers, because cocaine sensitization alters the expressions of HCN2 and HCN4 in different areas of mesocorticolimbic (MCL) system (Santos-Vera et al., 2019). To date, relation of HCN2 and HCN4 isoforms in the conservation of primary sequences among the four HCN channel isoforms should be further discussed. In short, these studies suggest that HCN channels can be regulated or remodeled under certain conditions, and these studies may help to expound the mechanisms of sympathetic nerve sprouting and remodeling. In the present study, although we have no further data to explain the mechanisms underlying the functions of HCN2 and HCN4 in PC12 cell differentiation, the pharmacological and molecular interference experiments suggest that HCN2 and HCN4 are involved in NGF-induced sympathetic neuron-like differentiation of PC12 cells, which may encourage future research in this field.

Regarding the electrophysiological phenotypes of PC12 cells, our patch clamp results show that the RPs of PC12 cells were about −65 to −70 mV, and NGF treatment did not significantly affect the RP. As for the excitability (ability to generate AP) of PC12 cells, our results are perplexing. It is likely that PC12 cells could be depolarized but did not show typical AP while with strong delayed rectification following the depolarization. The delayed rectification may be caused by the strong outward (K^+^) conductance as we show here. NGF-treated PC12 cells tended to show enhanced but incomplete “repolarization” compared with NGF-untreated PC12 cells (Figure 6B). These results are a bit similar with a study published 40 years ago (Dichter et al., 1977) which shows that NGF can increase the electrical excitability and acetylcholine sensitivity of PC12 cells. Alternative study shows that Na^+^ currents (TTX sensitive) were elicited in PC12 cells treated with NGF for 9 days (Yang et al., 2011). However, we could not induce the inward (Na^+^) current in the patch clamp study. The reasons may be that the PC12 cells have not developed mature functional voltage-gated Na^+^ channel in the present study, or, absence of other conductance blockade makes it difficult to accurately record Na^+^ currents. Overall, it is likely that the voltage-gated Na^+^ channel is immature and not a standard accessory of NGF-treated PC12 cells, as divergent results about the presence of voltage-gated Na^+^ channel emerges from different experiments. The evoked “depolarization” of PC12 cells in our study was likely not caused by voltage-gated Na^+^ channels but might be caused by HCN channels or alternative unknown inward currents, but our experiments cannot exclude the existence of voltage-gated Na^+^ channels in NGF-treated PC12 cells in a general sense. Here, HCN channel currents (Ih currents) were elicited both in NGF-untreated and NGF-treated PC12 cells and the currents were confirmed by three HCN channel blockers. These functional results further support our molecular results about the expression of HCN channels in PC12 cells.

A limitation of the present study is that we did not deeply investigate the signaling pathway by which HCN channels facilitate NGF-induced PC12 cell differentiation. Based on published literatures (Wainger et al., 2001; Vaudry et al., 2002; Zhong and Zucker, 2005; Liao et al., 2010; He et al., 2014; Möller et al., 2014; Nagai et al., 2016; Lee and MacKinnon, 2017; Sartiani et al., 2017), we suggest the potential signaling pathway as follows. Differentiation-stimulating factor(s) such as NGF induces production of cAMP in PC12 cells; cAMP activates Rap1 signaling pathway including its downstream MEK-ERK signaling by means of phosphorylation and thus promotes PC12 cell differentiation; cAMP can directly activate HCN channels via binding to the channel protein, and may also activate HCN channels via phosphorylation; in addition, Rap1 signaling can regulate the activity of HCN channels, and activated HCN channels in turn act on the Rap1 signaling pathway and thus regulate the differentiation of PC12 cells. Nevertheless, the roles of HCN channels in NGF-induced PC12 cell differentiation warrant further experimental study.

In summary, this study demonstrates that all the four HCN isoforms are expressed in PC12 cells. Specifically, HCN2 and HCN4, but not HCN1 and HCN3, play important roles in NGF-induced PC12 cell differentiation toward sympathetic neuron. In addition, NGF modulates the electrophysiological phenotypes of PC12 cells partially via affecting HCN channel activity. The study provides new information in understanding the mechanisms of neuronal differentiation and sympathetic nerve sprouting. These information may be helpful to the development of new strategies in the treatment and prevention of sympathetic nerve remodeling-related abnormalities including ventricular tachyarrhythmias in healing and healed myocardial infarction, cardiomyopathy, atrial fibrillation, autonomic dysfunctions and intricate neuropathic diseases.

## Data Availability

The datasets generated for this study are available on request to the corresponding authors.

## Ethics Statement

The cell use protocols were approved by the Ethics Committee of Southwest Medical University and were in accordance with the ethical standards laid down in the 1964 Declaration of Helsinki and its later amendments. The manuscript does not contain animal and clinical studies.

## Author Contributions

YW, X-RZ, and J-MC conceived and supervised the study. L-YZ and Z-JS performed the RT-qPCR experiments and Western Blot experiments. JL and NL cultured the PC12 cells. Y-CL and LW conducted the immunofluorescent staining experiments. X-RF and Z-CF made great contribution to the revision of the manuscript. YW, L-YZ, and Z-JS analyzed the data and wrote the manuscript draft. J-MC revised the manuscript.

## Conflict of Interest Statement

The authors declare that the research was conducted in the absence of any commercial or financial relationships that could be construed as a potential conflict of interest.
