# Supplementary material for: Hyperpolarization-Activated Cyclic Nucleotide-Gated Ion (HCN) Channels Regulate PC12 Cell Differentiation Toward Sympathetic Neuron
Source: Front Cell Neurosci. 2019 Sep 20;13:415. doi: 10.3389/fncel.2019.00415 (PMC6763607; doi:10.3389/fncel.2019.00415)
Supplement: Supplementary file 1 [file Table_1.docx]

**Supplementary materials**

**Hyperpolarization-activated cyclic nucleotide-gated ion (HCN) channels regulate PC12 cell differentiation towards sympathetic neuron**

Li-Ying Zhong^1,2^ **^†^**, Zhang-Jing Shi^1,2^**^†^**, Xin-Rong Fan^1^**^†^**, Zhong-Cai Fan^1^**^†^**, Jian Luo^1^, Na Lin^3^, Ying-Cai Liu^1^, Lin Wu^2,4^, Xiao-Rong Zeng^2^, Ji-Min Cao^5*^, Yan Wei^2*[
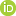
](https://orcid.org/0000-0002-2682-3780)^

RUNNING TITLE: HCN channels and neuronal differentiation

^1^ Department of Cardiology, The Affiliated Hospital of Southwest Medical University, Luzhou, China

^2^ Key Laboratory of Medical Electrophysiology of Ministry of Education and Medical Electrophysiological Key Laboratory of Sichuan Province, Collaborative Innovation Center for Prevention and Treatment of Cardiovascular Disease, Institute of Cardiovascular Research, Southwest Medical University, Luzhou, China

^3^ Department of Respiratory Medicine, Rongcheng People’s Hospital, Rongcheng, China

^4^ Department of Cardiology, Peking University First Hospital, Beijing, China

^5^ Key Laboratory of Cellular Physiology of Ministry of Education, Department of Physiology, Shanxi Medical University, Taiyuan, China

^*^ Correspondence: [weiyan.1111@swmu.edu.cn](mailto:weiyan.1111@swmu.edu.cn) (Y.W.), Tel: +86 830-3160619; [caojimin@126.com](mailto:caojimin@126.com) (J.C.), Tel: +86 351-4135246

**^†^** These authors contributed equally to this work.

**Table S1.** Nucleotide sequences of the siRNAs.

| Name | Sense Sequence (5’-3’) | Antisense Sequence (5’-3’) |
| --- | --- | --- |
| HCN1 | CCAAUCAACUAUCCUCAAATT | UUUGAGGAUAGUUGAUUGGTT |
| HCN2 | CCAUGCCGUUGUUUGCCAATT | UUGGCAAACAACGGCAUGGTT |
| HCN3 | CCACUUCAAUGCAGUCGUUTT | AAGCACUGCAUUGAAGUGGTT |
| HCN4 | GGUUCGGCCACUUUCAUAATT | UUAUGAAAGUGGCCGAACCTT |
| N.C. | UUCUCCGAACGUGUCACGUTT | ACGUGACACGUUCGGAGAATT |

HCN, hyperpolarization-activated cyclic nucleotide-gated channel. N.C., Negative control.

**Table S2.** Primers used in Q-PCR analysis.

| Gene | Forward Sequence (5’-3’) | Reverse Sequence (5’-3’) | Product size, bp |
| --- | --- | --- | --- |
| HCN1 | GCTGACATGCGCCAGAAGAT | CGCGTTAGCAAAGAGAGGCA | 165 |
| HCN2 | CGTGGTTCGTGGTAGACTTC | GCAGACTGAGGATCTTGGTG | 138 |
| HCN3 | CGTGGGCAGGAAGATGTAC | CCTTACACTGGCTGTTCTCC | 142 |
| HCN4 | ATTCTCGCTAAGGATGTTCGG | ATCGTCAGGTCCCAGTAAAATC | 123 |
| TH | GTGAACCAATTCCCCATGTG | CAGTACCGTTCCAGAAGCTG | 139 |
| GAP-43 | CGACAGGATGAGGGTAAAGAA | GACAGGAGAGGAAACTTCAGAG | 141 |
| GAPDH | TCCAGTATGACTCTACCCACG | CACGACATACTCAGCACCAG | 149 |

HCN, Hyperpolarization-activated Cyclic Nucleotide-gated Channels. TH, Tyrosine Hydroxylase. GAP-43: Growth associated protein-43. GAPDH, Glyceraldehyde-3-Phosphate Dehydrogenase.
